# Efficiency of reciprocating systems reciprocated at different angles in removing root-canals fillings with an MTA-type sealer: an Ex-vivo study

**DOI:** 10.1186/s12903-022-02390-0

**Published:** 2022-08-17

**Authors:** Ahmad A. Madarati, Aya M. N. Sammani, Ahmad A. Alnazzawi, Ali Alrahlah, Eugenio Pedullà

**Affiliations:** 1 Restorative Dental Sciences Department, College of Dentistry, Taibah University, Madina, Kingdom of Saudi Arabia; 2 Present Address: Riyadh (12252), Kingdom of Saudi Arabia; 3 Department of Substitutive Dental Sciences, College of Dentistry, Taibah University, Madina, Kingdom of Saudi Arabia; 4 Restorative Dental Sciences Department, College of Dentistry, King Saud University, Riyadh, Kingdom of Saudi Arabia; 5 Department of General Surgery and Surgical-Medical Specialties, University of Catania, Catania, Italy

**Keywords:** Endodontics, Micro-ct, MTA, Reciprocating, Removal, Retreatment, Sealer, Angle

## Abstract

**Background:**

There have been no reports on the impact of different reciprocating angles on retreatment performance of reciprocating files. This ex-vivo study compared the efficiency of three reciprocating systems in removing MTA-type sealer-based filling materials and investigated the influence of different reciprocating angles on their retreatment ability.

**Methods:**

140 root-canals were instrumented to a 35 apical size and filled with an MTA-type sealer and gutta-percha cones. Samples were scanned by micro-computed-tomography and the root-canals fillings volumes were measured. Samples were divided into 7 groups according to the reciprocating angles at which the *WaveOne-Gold* (*WOG*), *Reciproc-Blue* (*RB*) and *R-Motion* (*RM*) systems were reciprocated to remove the root-canals’ fillings. The *WOG-150/30, WOG-90/30, RB-150/30, RB-90/30, RM-150/30 and RM-90/30* groups in which the systems were reciprocated at 150/30 and 90/30 (counterclockwise/clockwise) angles. In the *RB-270/30* group the *RB* system was reciprocated at 270/30 angles. Samples were re-scanned and the remaining filling materials’ (RFMs) volumes were measured. The percentage of the RFMs volume and its mean value for each group were calculated and data were statistically analysed at 0.05 significance level.

**Results:**

The *WOG* system resulted in less RFMs (2.24%) when reciprocated at 90/30 angles compared to that resulted from the 150/30 angles (4.96%) [*P* = 0.002]. The *RB* system reciprocated at 90/30 angles resulted in less RFMs (2.67%) compared to that resulted from the 270/30 angles (6.64%) [*P* = 0.001]. The RFMs after using *RM* system reciprocated at 90/30 (6.02%) and 150/30 (7.61%) were greater than those of *WOG* (2.24 and 4.96%) and *RB* (2.67 and 4.34%) reciprocated at the same angles (*P* < 0.05)*.* The longest time required to remove the filling materials was recorded with the *RB-270/30* group (6.06 min) [*P* = 0.00]. The times required when the *WOG*, *RB* and *RM* files reciprocated at 90/30 angles (3.59, 3 and 3.05 mins, respectively) were shorter than those when files were reciprocated at 150/30 angles (5.25, 4.98 and 3.67 min, respectively) [*P* < 0.05].

**Conclusions:**

The *WOG* and *RB* systems removed more MTA-type sealer-based root-canals fillings than the *RM* system. Lower counterclockwise reciprocating angles improve the retreatment ability of reciprocating systems, especially the *WOG* system and can reduce the time required for retreatment procedures.

## Background

When periapical lesions develop or previously exist lesions do not heal, a non-surgical root-canal retreatment is usually considered to uncover the areas that harbor microorganisms or infected remnants. Consequently, the ability to be removed from root-canals is one main property of an ideal root-canals filling material [1]. While gutta-percha has been the gold standard core material, there has been no agreement on the superiority of one sealer over another. Calcium silicate-containing sealers (CSCSs) were introduced almost 10 years ago and have been intensively investigated. Though ex-vivo studies have shown good properties, clinical long-term success has not been confirmed. Recent clinical studies, with short-term follow-ups, have shown promising outcomes [2, 3]. Once they set, CSCSs are hard, create hydroxyapatite crystals that bond to dentine and can penetrate the dentinal tubules [4]. As a result, the ability to retreat root-canals filled with CSCSs-based fillings and gaining patency to the root-canals’ terminus, to properly disinfect the root-canals system, is one main concern. Ex-vivo studies have shown conflicting results in this regard as well as regarding the time required to perform retreatment procedures [4–11].

Sfeir et al. [12] pointed out the need for further studies that better simulate retreatment clinical conditions to avoid methodological biases. However, studies investigated retreatability of CSCSs as well as other types of sealers confirmed that there are neithr methods nor techniques that can completely remove old root-canals’ filling materials. Consequently, there is always need for better instruments and supplementary methods. Additional agitation of intra-canals’ irrigants by laser irradation, sonic or ultrasonic vibrations has been reported to significantly improve removal of fillings materials [13–15]. *Reciprocating* systems have also shown good results [8, 16–19]. Madarati et al. found that reciprocating instruments were better than rotary instruments in removing root canals fillings [16]. The *R-Motion* system (FKG Dentaire, Switzerland) is a new nickel-titanium single-file *reciprocating* system with a non-cutting tip and a triangle-based symmetrical cross-section shape. The only available study showed that this system had a similar shaping ability of curved root-canals to that of *Reciproc-Blue*, *HyFlex-CM* and *XP-shaper *systems [20]. *Reciprocating* systems are usually used at different reciprocating angles. Most of these systems engae with dentine when rotate in the *counterclockwise* direction and cut dentine when rotate in the *clockwise* direction. Consequently, the degree of the *clockwise* or *counterclockwise* rotations may influence the cutting effeciency or shaping ability of these instruments. While there are only two studies that investigated this aspect [21, 22], to the best of our knowledge there have been no reports on the impact of different reciprocating angles on the retreatment ability of *reciprocating* systems. In addition, the retreatment ability of *R-Motion* (*RM*) system has not been invistigated yet. The authors believed that this knowledge’s gap is important and needs to be addressed.

Different methods were used to measure the RFMs after retreatment procedures. Three-dimensional (3-D) analysis, by micro-computed tomography (micro-CT), is accurate to great extent and more accepted among researchers [23]. Therefore, the current study aimed at: (a) comparing the efficiency of three *reciprocating* systems, *WaveOne-Gold* (*WOG*), *Reciproc Blue (RB)*, and *R-Motion* (*RM)*, in removing filling materials with an MTA-type sealer (EndoSeal, Maruchi, Korea), and (b) investigating the influence of different reciprocating angles on retreatment ability of these *reciprocating* systems using the micro-CT method. The main two null hypotheses tested were:

- There would be no significant differences among the reciprocating systems in removing root-canals’ fillings with an MTA-type sealer.
- There would be no significant differences in retreatment ability of each system when reciprocated at different reciprocating angles.

## Methods

### Ethical approval & teeth collection

The study was approved by the National Science Technology and Innovation Plan’s ethical committee (Saudi Arabia) (Ref: 13-BIO57-05). It was conducted in full accordance with ethical principles, including the World Medical Association Declaration of Helsinki (version 2008). Upper premolars with two separated root-canals were collected from pools of teeth that were extracted for orthodontic reasons or periodontal diseases (NOT for this study) from governmental sector clinics in the Western Province, Saudi Arabia. Teeth with previous RCTs, dentine pins, caries, coronal restorations, fractures or cracks, resorptive defects, had root-canals curvature angles greater than 30 degrees according to the Schneider’s method [24], or had extra root-canals were excluded.

### Samples’ preparation

The teeth were sectioned 2 mm above the cementoenamel junction and the root-canals were cleaned and shaped with the 2-Shape rotary system (Micro-Mega, France) up to size 35. During root-canals’ instrumentation, each canal was irrigated between instruments with 1 ml of 5.25% sodium hypochlorite (NaOCl) (total of 10 cc). Upon instrumentation, root-canals were irrigated with 1 ml of 5.25% NaOCl activated sonically by the Medium Activator Tip (red) (DentsplySirona, Switzerland) mounted on the EndoActivator handpiece (DentsplySirona) for 30 s, then with 1 ml saline followed by 1 ml of 17% EDTA activated sonically for 30se. The residual irrigant was removed with a final rinse of 1 ml distilled water. The root-canals were filled with EndoSeal MTA sealer (Maruchi, Korea) and gutta-percha cones (Meta Biomed, Korea) using the modified hot technique. Two millimeters of the premixed sealer were injected to into each root-canal, then the tip of gutta the percha master cone was coated by the sealer and then inserted to the root-canal’s full working length. The modified hot technique was performed using the Buchanan System-B Pluggers and Element-Free obturation system (Kerr Cor, CA, USA) during which the heat source was adjusted at 120 °C [25]. The quality of the root-canal fillings was radiographically evaluated. After inserting temporary coronal restorations, samples were re-examined under microscopic magnification to check the integrity of the roots and then they were incubated at 37 ± 1 °C and 100% relative humidity. The study’s sample size yield 70 premolars with 140 root-canals; 20 root-canals for each study’s group (samples size calculation suggested 15 samples in each group considering 90% power calculation to detect differences when standard deviation is 3.950). This sample size was greater than what was reported in most of previous studies [18, 26].

### Groups’ sampling

The study included 7 groups according to the reciprocating systems and the different reciprocating angles that were adopted. Using the Google Random Number Generator (www.google.com), specimens were randomly allocated to the 7 equal groups (*n* = 10, each specimen had 2 root-canals). Each specimen was given a number from 1 to 70, then each number suggested by the Google Random Number’s generator was allocated to the groups from 1 to 7, consecutively. The mean volumes of the initial root-canals fillings (overall mean 6.11 mm^3^) did not significantly differ among the 7 groups (*P* > 0.05), which confirmed the random sampling.

### First micro-CT scannings, reconstructions & measurements of the root-canals fillings volumes

Specimens were placed in the scanning chamber of the SkyScan-1173 high energy Micro-CT machine (BrukerSkyScan, Belgium) in a certain pre-marked position (orientation) to enable the same position during the second scanning of specimens. After adjusting the appropriate parameters (675 ms exposure time, 36.8 µm image-pixel size, 0.5 mm brass-filter, 0.4 rotation-step for 360° angle, frame-average of 4, and 8 random-movement), samples were scanned by the SkyScan-1173 Micro-CT machine (BrukerSkyScan, Belgium). A flat-field correction was performed before scanning procedures to correct variations in the camera pixel sensitivity. Using the ©N-Recon software version 1.6.9.4 (BrukerSkyScan), the projected images were reconstructed to produce 2-dimensional (2-D) cross-sectional images of the samples’ inner structure. A 5 ring artifact reduction, 25% beam hardening compensation, and 2 smoothing using Gaussian kernel were applied. The reconstructed images were loaded to the Data-viewer software (version 1.5.6.2) (BrukerSkyScan) to determine images’ quality, reorient, resize, and to enable more accurate positioning and visual inspection. The registration data-set was saved and loaded in the ©CTAn software (version 1.20.8.0) (BrukerSkyScan) to analyse images selectively and then to measure the volume of the root-canal fillings.

### Removal attempts of the root-canals filling materials

The 7 study’s groups were according to the reciprocating angles at which the three reciprocating systems were reciprocated to remove the root-canals’ filling materials as follows:

#### WOG-150/30 and WOG-90/30 groups

In which the fillings materials were removed using the *WaveOne-Gold* size 35 *reciprocating* files (DentsplySirona, Switzerland) reciprocated at 150/30 and 90/30 (counterclockwise/clockwise) angles, respectively.

#### RB-150/30, RB-90/30 and RB-270/30 groups

In which the filling materials were removed using the *Reciproc-Blue* size 40 *reciprocating* files (VDW, Munich, Germany) reciprocated at 150/30, 90/30 and 270/30 (counterclockwise/clockwise) angles, respectively.

#### RM-150/30 and RM-90/30 groups

The *R-Motion* size 40 *reciprocating* files (Switzerland) were used at 150/30 and 90/30 angles, respectively.

Each reciprocating file was used at 350 rpm and 3.5 N/cm^2^ for removal of the filling materials of 2 root-canals, which were irrigated with 1 mm of 5.25% NaOCl after each file insertion during the retreatment procedures (with total of 5 mm). The residual irrigant was removed with a 1 mm final rinse of distilled water. The removal attempts of the filling materials were deemed when the root canals’ working length were reached with the endodontic files five times and no filling materials were found on the files [27]. The time, in minutes, to complete the filling materials removal attempt and associated complications (such as root perforations, ledge formation or fracture of instruments, if any) were recorded.

### Second micro-CT scannings, reconstructions & measurements of the RFMs volume

Samples were placed within the micro-CT machine’s chamber in the same positions and orientation as in the first Micro-Ct scanning. The parameters of Data-viewer software (1.5.6.2) (BrukerSkyScan) used in the first scanning also enabled better repositioning. Samples were then scanned using the SkyScan 1172 machine with the same parameters used in the first Micro-Ct scanning. Also, the reconstructions (using the NRecon software) and measurements of the volume of the RFMs (using the CTan software) were performed the same way for measuring the volume of the initial filling materials volume.

### Calculation of the RFMs and statistical analysis

The percentage of the RFMs volume after retreatment procedures’ attempts was calculated by the following equation [28]:

$$\frac{Volume\;of\;remaining\;filling\;material}{{Volume\;of\;original\;filling\;material}} \times 100 = Volume\left( \% \right)of\;remaining\;filling\;material\left( {RFM} \right)$$

Data were entered into SPSS software version 20 (SPSS Inc, Chicago, IL) to calculate the mean of the percentage of the RFMs volume for each group. The Sapiro-wilk normality test showed a normal distribution of data (*P* > 0.05). Therefore, Independent Samples-T, one-way ANOVA and two-way ANOVA statistical tests were used at 0.05 level of significance.

## Results

The mean values of the RFMs percentage and the time required to remove the fillings materials are presented in Table 1. Overall, the lower the *counterclockwise* reciprocating angle, the less the RFMs after removal attempts (*P = *0.045). The *WOG* system resulted in significantly less RFMs (*P* = 0.002) when reciprocated at 90/30 (*counterclockwise/clockwise*) angles (2.24%) compared to 150/30 angles (4.96%). Both the *RB* and *RM* systems resulted in less RFMs when reciprocated at 90/30 angles (2.67 and 6.02% respectively) when compared to the 150/30 angles (4.34 and 7.61%, respectively) but the differences were not significant (*P* = 0.073* and *0.069). Only when the *RB* system was reciprocated at 90/30 angles, the RFMs (2.67%) were significantly less than that resulted from the 270/30 reciprocating angles (6.64%) [*P* = 0.001]. The RFMs obtained after using the *RM* system when reciprocated at 90/30 and 150/30 angles (6.02 and 7.61%, respectively) were significantly greater than those caused by the *WOG* (2.24 and 4.96%) and *RB* (2.67 and 4.34%) systems when reciprocated at the same angles (*P* < 0.05)*.*

**Table 1:** Mean & standard deviation of the remaining filling material volume (%) and Time required for attempt at removing the filling material (min) using systems at different reciprocating angles

Variables	Reciprocating systems groups						
	WaveOne-Gold		Reciproc-Blue			R-Motion	
	150/30 (*N* = 20)	90/30 (*N* = 20)	150/30 (*N* = 20)	90/30 (*N* = 20)	270/30 (*N* = 20)	150/30 (*N* = 18)	90/30 (*N* = 20)
RFM volume (Mean ± SD %)	4.96 ± 3.11	2.24 ± 1.78	4.34 ± 3.31^a^	2.67 ± 1.93^b^	6.64 ± 3.98^b,c^	7.61 ± 2.65	6.02 ± 2.59
Significance	*P* = 0.002		*P* = 0.073		*P* = 0.001	*P* = 0.069	
	*P* < 0.05						
Filling removal time (min)	5.25 ± 0.94	3.59 ± 0.87	4.98 ± 0.83^a^	3.00 ± 0.46^a,b^	6.06 ± 1.24^a,b^	3.67 ± 0.75	3.05 ± 0.54
Total	*P* = 0.000		*P* = 0.002			*P* = 0.007	

Symmetrical letters indicate a significant different between paired groups (*P* < *0.05*)

Overall, the lower the *counterclockwise* reciprocating angle, the shorter the time required to remove the filling materials (*P* = 0.045). The longest time was recorded with *RB* files when reciprocated at 270/30 angles (6.06 min) [*P* = 0.00]. The times required to remove the filling materials when the *WOG*, *RB* and *RM* files reciprocated at 90/30 angles (3.59, 3 and 3.05 min, respectively) were significantly shorter than those required when they were reciprocated at 150/30 angles (5.25, 4.98 and 3.67 min, respectively) [*P* < 0.05].

## Discussion

The results of this study showed significant differences among the reciprocating systems in removing root-canals’ fillings with an MTA-type sealer. Also, they revealed significant differences in retreatment ability of some systems when reciprocated at different reciprocating angles. Therefore, both null hypotheses were rejected.

The ability to be removed from root-canals systems is one of the main criteria of root-canals filling materials if root-canals retreatment is considered. Studies showed conflicting results regarding the retreatability of CSCSs and traditional sealers [4, 5, 8]. Nevertheless, they also showed that retreatability of CSCSs is no longer a main concern, which may explain their improved acceptance as sealers in daily endodontics. It is important to mention that all root-canals (100%) in the current study deemed patent after retreatment procedures. Therefore, our results were comparable to those obtained in previous studies and confirmed the retreatability of these types of sealers.

We did not use solvents in the current study, which maybe considered as a limitation. However, their role in enhancing removal of root-canals filling materials is still debatable [29, 30]. Moreover, Horvath et al. pointed out that solvents can result in blockage of dentinal tubules by gutta-percha and sealers and suggested using solvents only whenever the root-canals’ working length could not be reached [31]. Previous studies found that solvents reduced the time to reach the working length but didn’t improve root-canals’ cleanliness [29]. Interestingly, solvents’ extrusion was reported to be below the permissible toxic dose, hence the risk to patients can be negligible [32]. Nevertheless, future studies are needed to investigate the long-term safety of solvent’s applications.

It is claimed that teeth decoronation results in better standardization of the working length and filling-materials’ removal and facilitates accessing the root-canal system [33, 34]. However, even when the crown presents, standardization can still be accomplished by standard working lengths and standard access cavities. In addition, the impact of the limited access to root-canals on the effectiveness of instruments retreatment ability should not be overlooked, because clinically teeth undergoing retreatments may have crowns. Teeth in the current study were sectioned 2 mm above the cementoenamel junction, which maximized standardization. In addition, this still reflects the clinical situations, in which retreatment is usually performed on heavily destructed teeth. However, the impact of decoronating on retreatment ability of rotary systems needs to be addressed by further research works.

Different methods have been implemented to assess the retreatment ability of different rotary files. Two-D assessment methods have some drawbacks which may limit their acceptance [33, 35–37]. The 2-D radiographs, for example, provide only 2-D information and may show some distortions of the 3-D structures and cannot visualize small volumes of debris [35, 38]. Three-D assessment using cone-beam computed tomography (CBCT) or Micro-CT are non-destructive and enable more accurate 3-D measurements with different interventions [23]. It might be argued that it was better to scan samples before instrumentation to confirm groups’ sampling standardization and could be one limitation. Such step is fundamental when investigating aspects of root-canals instrumentation (i.e. centering ability of rotary instruments). However, the current study measured the changes of the root-canals’ fillings volume after retreatment procedures to determine which instrument and what reciprocating angles performed better. Therefore, it was important to standardize volumes of the initial root-canals fillings, which was achieved (there were no significant differences among the study groups regarding the volume of the initial root-canals fillings). Retreatment procedures were performed by the *RB* and *RM* files size 40 and *WOG* system size 35*.* Using different sizes, maybe considered as bias and a limitation of this study. However, our un-published data showed that different sizes and tapers of *rotary* and *reciprocating* files did not affect their retreatment ability. In fact, they showed that instruments of smaller sizes were more effective than those of larger sizes.

This study investigated the retreatment efficiencies of *WOG, RB* and *RM* files reciprocated at different angles in removal of root-canals’ fillings with an MTA-type sealer. Removal of filling materials usually enables instruments and irrigants to reach more areas of the root-canals system, which improves its disinfection [18]. To this date there has been no retreatment protocol that can completely remove the root-canals fillings, hence some RFMs following retreatment procedures is inevitable [14, 23]. The current study also showed that none of the reciprocating systems, regardless the reciprocating angles, was able to remove the MTA-type sealer-based fillings. It is generally accepted that even the most sophisticated endodontic files cannot entirely instrument root-canals walls [39]. Therefore, implementing additional irrigation/agitation and advanced techniques is paramount. Supplementary irrigants agitation by sonic or ultrasonic vibrations or laser irradiation significantly improved removal of root-canals fillings [13–15]. In addition, there is always need to improve the retreatment efficiency of instruments. The ability of various nickel-titanium systems, including reciprocating ones have been investigated [27, 40]. However, to the best of our knowledge, the present study is the first one that compared different reciprocating angles during root-canals retreatment and the first one that tested the *RM* system retreatment ability. Overall, the lower the *counterclockwise* reciprocating angle, the less the RFMs after removal attempts (*P* = 0.045), hence the null hypothesis was rejected. The *WOG* and *RB* systems were more effective in removing the root-canals fillings with EndoSeal MTA sealer when reciprocated at 90/30 (counterclockwise/clockwise) angles than 150/30 or 270/30 ones, respectively. In particular, the 90/30 reciprocating angles required a shorter time than the 150/30 or 270/30 ones to remove the filling materials. These results could be due to the greater reciprocating cycles performed by the instruments. When the instruments are reciprocated at 90/30 angles, they make a 60° turn for every cycle. Whereas when the instruments are reciprocated at 150/30 angles, they do a 120° turn, which means that greater number of reciprocating cycles are required to complete one full rotation. More precisely, one whole rotation is completed after 6, 3 or 1,5 reciprocating cycles when the angles are set at 90/30, 150/30 or 270/30 degrees respectively. It means that the tested instruments performed more reciprocating cycles at 90/30 reciprocating angles considering they were used at the same speed (350 rpm). Two previous studies reported that greater number of cycles (lower reciprocating angles) resulted in superior cutting efficiency [21, 22]. However, performance of *rotary* and *reciprocating* files in preparing root-canals without filling materials is most probably different from that when they are used for removing root-canals fillings. More importantly, the lack of reports in the literature necessitates further investigations of wider range of reciprocating angles of different systems for different parameters (i.e., debris extrusion and the centering ability) to draw proper clinically relevant conclusions.

Nevertheless, the RFMs percentage was significantly less in the 90/30 reciprocating angles groups only for the *WOG* system compared to the 150/30 degrees and the *RB* system compared to the 270/30 degrees. This difference between the two systems could be due to the different instruments designs and different cutting efficiencies. The *RB* file has S-shaped cross-section and two cutting edges, whereas the *WOG* has a parallelogram-shaped cross-section with 1 or 2 alternating cutting edges. Therefore, the higher number of reciprocating cycles performed at 90/30 degrees increased the filling materials’ removal significantly for the *RB* and *WOG*; notably the latter reached the significance when was compared with the 150/30 degrees. On the other hand, there was a significant difference when the *RB* files reciprocated at 90/30 degrees compared to 270/30 degrees, because the number of reciprocating cycles were 6 and 1,5 respectively, hence there was a greater difference in the effectiveness of fillings’ removal. Studies have reported inconsistent effectiveness of *reciprocating* and *rotary* systems in removing filling materials [8, 16, 17, 23, 27, 33, 38, 41]. This inconsistency can be explained by the different methodologies such as using plastic blocks or extracted teeth, the quality and types of initial root-canals fillings, different angles and shapes of the root-canals, supplementary retreatment procedures, and assessment’ methods. Some studies reported similar effectiveness of *reciprocating* and *retreatment rotary* systems [8, 16]. The results of our unpublished data were consistent with some studies and showed that the *reciprocating* systems were more effective than *rotary* systems [17, 27]. Madarati et al., pointed out that the reciprocating motion was the main reason for the good retreatment ability of the *reciprocating* files [16]. The alternating motion (counterclockwise/clockwise) of the reciprocating files may dislodge the root-canals filling materials, especially the hard MTA-type sealer, from the root-canal walls, facilitating its removal coronally. Also, the retreatment ability of the reciprocating systems can be due to their good centering ability [33]. However, both of *WOG* and *RB* systems were superior to the *RM* system, which can be explained by the different instruments’ designs. The three systems have different number of contacts of the cutting-edges with root-canals’ walls. Unlike the *RB* file which has an S-shape cross-section with two contact edges, the *RM* file has a rounded-triangular symmetrical cross-section with three contact cutting-edges. The *WOG* file has a parallelogram cross-section shape with four cutting-edges. However, it alternately contacts root-canals’ walls only with 1–2 contact cutting-edges, depending on the location along the file (www.dentsplySirona.com). It seems that less contact cutting edges increases the retreatment ability. Nevertheless, further investigations of the impact of more different reciprocating angles on the retreatment ability of reciprocating instruments can explore more aspects and enables better conclusions.

Overall, the lower the *counterclockwise* reciprocating angle, the shorter the time required to remove the filling materials (*P* = 0.045); with the longest time recorded with the *RB* files reciprocated at 270/30 angles (6.06 min). The times required to remove the filling materials when the *WOG*, *RB* and *RM* files reciprocated at 90/30 angles (3.59, 3 and 3.05 min, respectively) were significantly shorter than those required when these files were reciprocated at 150/30 angles (5.25, 4.98 and 3.67 min, respectively). Shortest reciprocating angles, which means more cycles of rotation, may facilitate penetration of the root-canals fillings’ core. In a study conducted very recently (unpublished data) the operator noticed that the *rotary* systems which were faster during the initial penetration into root-canals fillings needed the shortest times of retreatment procedures. However, it was found that easier penetration into the root-canals’ fillings did not result in better retreatment efficiency. The reciprocating systems, which needed longer removal times, resulted in less RFMs. Unlike those unpublished results, the current study results indicate that the shorter the time for fillings removal attempts, the less the RFMs. These findings are inconsistent with those of a previous study [5]. Obviously what applies for *reciprocating* systems may not apply for *rotary* ones. Nevertheless, there is inconsistency in the literature regarding the time needed for retreatment procedures when different endodontic instruments were used. While Jorgenen et al. found that *retreatment rotary* files were faster than *reciprocating* ones [42], other studies reported no differences between them [16, 38, 41]. On the other hand, Zuolo et al. found that the *Reciproc* system was faster than the *Mtwo-R* one [40]. This conflicting literature can be due to different research methodologies. Nevertheless, clinicians better stress on measures that enhance removal of the filling materials to improve disinfection of the root-canals system rather than overvalue the time needed for retreatment procedures.

## Conclusions

Within the conditions and limitations of the current study, the following can be concluded:

- The *WOG* and *RB* systems were better in removing root-canals fillings with an MTA-type sealer than the *RM* system.
- Less *counterclockwise* reciprocating angles could improve the retreatment ability of *reciprocating* systems, especially the *WOG* system when removal of fillings with an MTA-type sealer was attempted.
- Also, less *counterclockwise* reciprocating angles could reduce the time required for retreatment procedures of fillings with an MTA-type sealer.

## Data Availability

The data that support the findings of this study are available from the National Science Technology and Innovation Plan (KSA) (https://npst.ksu.edu.sa/en) but restrictions apply to the availability of these data, which were used under license for the current study, and so are not publicly available. Data are however available from the authors upon reasonable request and with permission of the National Science Technology and Innovation Plan (KSA).

## Abbreviations

RCTs: Root-canals treatments
CSCSs: Calcium silicate-containing sealers
RFMs: Remaining fillings materials
CT: Computed tomography
3-D: Three dimensional
2-D: two dimensional
WOG: WaveOne gold
RB: Reciproc blue
RM: R-motion
